# Imaging Through Scattering Tissue Using Near Infra-Red and a Convolutional Autoencoder

**DOI:** 10.3390/s26082507

**Published:** 2026-04-18

**Authors:** Alon Silberschein, Amir Shemer, Chanan Berkovits, Yair Engler, Ariel Schwarz, Eliran Talker, Yossef Danan

**Affiliations:** 1 Department of Electrical and Electronics Engineering, Ariel University, Ariel 4070000, Israel; 2 Department of Electrical and Electronics Engineering, Azrieli College of Engineering, Jerusalem 9103501, Israel

**Keywords:** tumor margin detection, near-infrared imaging, halogen light source, convolutional neural network, autoencoder, subsurface imaging, biomedical imaging, deep learning

## Abstract

Accurate delineation of tumor margins is critical for complete resection and minimizing recurrence, yet existing imaging modalities such as MRI, CT, and fluorescence imaging suffer from limitations including high cost, limited accessibility, and intraoperative constraints. In this study, we propose a low-cost, non-invasive approach for subsurface imaging based on near-infrared (NIR) illumination combined with deep learning. A controlled experimental setup was developed in which structured patterns displayed on an electronic paper screen were concealed beneath a tissue-mimicking chicken phantom and imaged using a NIR-sensitive camera under halogen illumination. A convolutional autoencoder based on a U-Net architecture was trained on approximately 10,000 paired samples to reconstruct hidden structures from highly scattered surface images. The proposed method achieved strong reconstruction performance, with the best model reaching a peak signal-to-noise ratio (PSNR) of 20.14 dB, structural similarity index (SSIM) of 0.92, and feature similarity index (FSIM) of 0.94, significantly outperforming conventional Wiener filtering. Qualitative results demonstrated accurate recovery of subsurface shapes with minor smoothing artifacts. While generalization to out-of-distribution samples remains limited, the findings highlight the potential of combining NIR imaging and deep learning for safe, rapid, and cost-effective subsurface visualization. This work establishes a foundation for future development toward clinically relevant tumor margin detection.

## 1. Introduction

The accurate detection of clear tumor margins is essential for achieving complete tumor removal and reducing the risk of recurrence [1]. When tumors are located near critical neurological structures, margin detection becomes even more important, as excessive tissue removal may endanger vital organs or nerve tissues [2]. Tumor margins are typically assessed using MRI, CT, and targeted fluorescence imaging [1,3,4,5]. However, these techniques have notable limitations. MRI and CT are susceptible to intraoperative tissue shifts, reducing spatial accuracy, while fluorescence imaging is limited by complex photophysical processes and risks such as phototoxicity and photobleaching [1,6,7]. In addition, MRI and CT systems are expensive, require specialized personnel, and are not widely accessible. Some of these modalities also rely on ionizing radiation or involve other harmful side effects, raising concerns regarding patient safety [6,7]. Ultrasound mammography is also widely used but is inherently limited in resolving fine tissue characteristics. More advanced approaches, such as photoacoustic imaging, elastic scattering spectroscopy, and Raman spectroscopy, offer improved contrast and molecular sensitivity. However, these techniques face notable challenges, including limited spatial resolution and relatively slow acquisition speeds, which hinder their routine clinical adoption [3].

A promising alternative is near-infrared (NIR) imaging, offering a safe, non-invasive approach for detecting subsurface structures. Unlike X-ray–based imaging, NIR radiation is non-ionizing and therefore harmless to biological tissues. NIR imaging is effective for biological tissue visualization due to reduced scattering, reflection, and absorption at longer wavelengths. Additionally, tissue absorption is lower in the NIR range compared to visible light, which reduces surface glare and improves the visibility of subsurface structures [8].

Human tissue exhibits dispersive optical properties that differ from denser tumor tissue, leading to increased reflectance in tumor regions under broadband halogen illumination [9]. Standard silicon-based cameras are optimized for the visible range and have limited sensitivity to NIR wavelengths [10]. While visible light is strongly scattered and absorbed, NIR light can penetrate up to ~2 cm into tissue, enabling subsurface visualization of tumors and blood vessels [11]. Combined with data-driven methods, NIR imaging has emerged as an effective approach for biomedical applications [10,11].

Compared to conventional modalities such as MRI and CT, NIR systems are more cost-effective and easier to deploy, while also avoiding ionizing radiation; additionally, in contrast to emerging optical techniques, NIR imaging can offer faster acquisition with simpler instrumentation. These advantages make NIR a promising complementary approach for biomedical imaging applications.

In this study, halogen lamps were used as the illumination source. These incandescent lamps provide a broad-spectrum output, with most of their energy in the NIR region. Only about 15–20% of their emission lies in the visible spectrum (400–700 nm), and less than 1% falls within the ultraviolet range [12,13]. As a close approximation of a black-body radiator, a halogen bulb emits radiation following Planck’s law, making it well suited for this imaging application [14].

Despite reduced scattering and absorption, NIR images acquired through tissue remain degraded, often exhibiting low contrast and distortion. Machine learning methods can address this limitation by delineating the mapping between scattered inputs and underlying structures, enabling reconstruction of subsurface features. Combined with NIR imaging, these approaches can transform ambiguous data into clinically useful information.

Recent advances in Artificial Intelligence (AI) have transformed the field of medical imaging including NIR imaging. Deep learning techniques enable the detection of complex patterns within large imaging datasets, substantially improving diagnostic accuracy and efficiency [15,16]. AI has already demonstrated success in analyzing CT, MRI, and ultrasound data for lesion evaluation and surgical planning, enhancing precision and clinical outcomes [16]. Methods based on convolutional neural networks (CNNs), spectral–spatial architectures, self-supervised learning, and transformer-based models have substantially advanced capabilities for classification, anomaly detection, and target localization in NIR images [17]. When combined with computational methods such as fusion imaging [18], K-nearest neighbor clustering [19], support-vector machines [19], or autoencoder-based neural networks [20,21], NIR imaging has been shown to significantly improve object detection through scattering tissue. However, existing AI systems for NIR imaging remain limited by the availability of sufficiently large and diverse datasets [21].

This study introduces a low-cost and efficient method for processing NIR images through scattering tissue, paired with a novel experimental setup for generating controlled, repeatable subsurface patterns to support model development. Our approach highlights the unique advantages of NIR as a safe, non-ionizing modality, while leveraging the power of deep learning to achieve reliable subsurface imaging.

## 2. Materials and Methods

### 2.1. Theoretical Overview

In this study, the acquired images were used as inputs to a CNN with an autoencoder architecture, designed to model the complex mapping between surface images affected by tissue scattering and the corresponding underlying structures. The network uses convolutional filters to scan across the images, efficiently capturing hierarchical spatial features, from low-level patterns such as edges and textures to higher-level structural representations, which are essential for reconstructing meaningful information from highly scattered and low-contrast input images [22,23]. The autoencoder can be expressed as:

(1) $$\tilde{x_{i}}=f(h_{i})=f(g\left(x_{i}\right))$$

where *x* is the input, *g* is the encoder function, and *f* is the decoder function. In this study, we adopted the U-Net architecture commonly used for medical image processing [24,25]. The U-Net was selected due to its proven effectiveness in medical image reconstruction tasks, particularly in scenarios with limited data. Its encoder–decoder structure with skip connections enables the preservation of spatial information while capturing higher-level features, making it well-suited for reconstructing images degraded by scattering.

The encoder consists of five sequential convolutional blocks, each comprising two convolutional layers with ReLU activation and batch normalization, followed by spatial down-sampling via max-pooling. As spatial resolution decreases, the number of feature channels increases from 64 to 768, enabling progressively more abstract feature extraction.

At the bottleneck, two additional 768-channel convolutional layers refine the highest-level features. The decoder mirrors the encoder through progressive up-sampling stages, incorporating skip connections that add corresponding encoder feature maps to preserve spatial information lost during pooling. Each decoding stage applies convolutional layers and batch normalization after up-sampling, gradually restoring the original spatial resolution.

**Figure 1:**
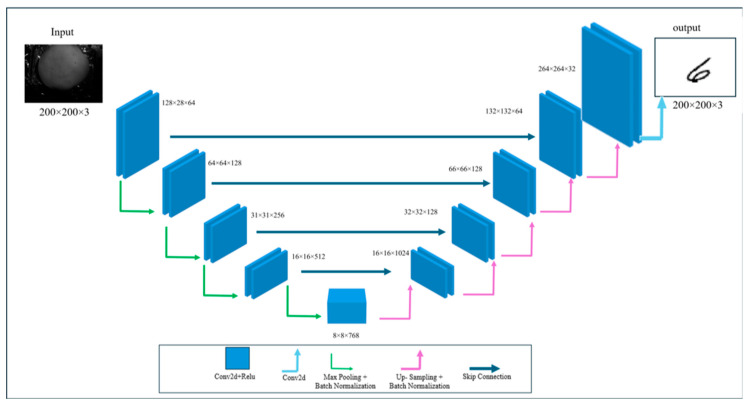
The diagram of the CNN used for our experiment.

The network concludes with a final convolutional layer using sigmoid activation to produce a reconstructed output image. The architecture of the convolutional autoencoder employed in this study is illustrated in Figure 1.

To ensure that the chosen architecture is well-suited to the task, we evaluated additional U-Net variants with different bottleneck sizes and reduced depth. This comparison was performed to verify that the selected model does not suffer from insufficient capacity or unnecessary complexity and represents a balanced trade-off between reconstruction performance and model size. Two additional U-Net variants were used for comparison: one with the same architecture but 1024 channels in the bottleneck, and another with the same architecture but a bottleneck of 512 channels and only two layers in both the encoder and decoder, to evaluate the impact of these structural modifications on performance.

### 2.2. Experimental Setup

Our experimental setup was designed to acquire subsurface optical images of a tissue-mimicking phantom while concealing structured patterns beneath it. The imaging system consisted of a monochrome industrial camera (Basler acA1440-220um, Basler AG, Ahrensburg, Germany) with a spatial resolution of 1440 × 1080 pixels. The camera was equipped with a 780 nm bandpass optical filter, enabling NIR imaging while suppressing out-of-band visible light.

**Figure 2:**
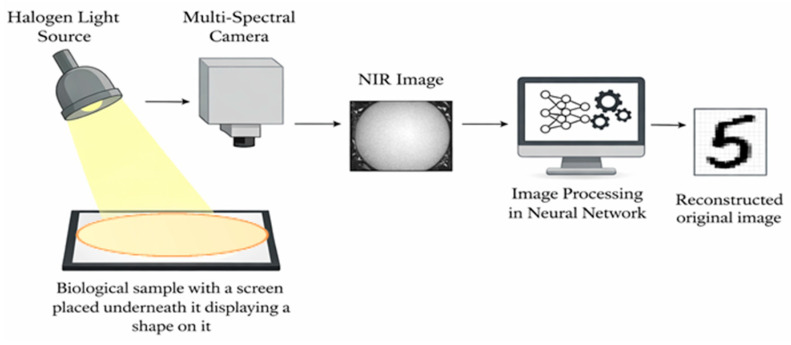
The schematic sketch of the experimental setup.

Beneath the phantom, an electronic paper (E-paper) display (Waveshare 4.3-inch e-Paper Display, Shenzen Waveshare Electronics Co., Ltd., Shenzhen, China) with a resolution of 800 × 600 pixels was positioned to present high-contrast black-and-white digit patterns. The reflective, non-emissive nature of the E-paper display minimized self-emitted light, ensuring that the displayed patterns influenced the recorded images only through light transmitted and scattered by the overlying phantom. The camera was positioned perpendicular (90°) at a fixed distance of 40 cm above the phantom. A schematic illustration of the complete setup is shown in Figure 2.

Illumination was provided by a broadband halogen light source with approximately 20 W power intensity positioned at an angle of 45° and a height of 24.5 cm relative to the phantom surface. This oblique illumination geometry was selected to reduce specular reflections and enhance subsurface scattering contrast. All components of the experimental setup—including the light source, phantom, E-paper display, and camera—were housed within a fully enclosed, light-tight box to prevent external ambient light from entering the system and to ensure stable and repeatable illumination conditions.

Heat generation from the light source was not actively suppressed; minor thermal variations were considered acceptable and may have contributed to increased variability in the training data.

The tissue-mimicking phantom consisted of a chicken breast sample cut to an approximate thickness of 5 mm. Chicken breast tissue was selected due to its optical scattering and absorption properties in the NIR spectral range, which are similar to those of human soft tissue [10].

During data acquisition, different shapes were sequentially displayed on the E-paper screen. Both the E-paper display and the camera were connected to a computer and controlled by a custom Python (version 3.12) script, which synchronized the presentation of each digit with image acquisition. Whenever the displayed digit was updated on the E-paper screen, the script automatically triggered the camera to capture a corresponding surface image of the phantom. This automated procedure ensured a one-to-one correspondence between each hidden digit and the observed surface scattering pattern, while maintaining consistent timing and acquisition conditions across the dataset. This acquisition procedure generated a paired dataset consisting of two complementary components: (1) the original black-and-white digit images obtained from the MNIST (Modified National Institute of Standards and Technology) database, and (2) the corresponding surface images of the phantom recorded while each digit was displayed beneath it.

In total, a dataset of approximately 10,000 paired image samples was used to train a convolutional autoencoder neural network. The network was trained using a batch size of 25, with the surface images of the phantom provided as input and the corresponding digit images serving as target outputs. Training was performed on an NVIDIA RTX A5000 (NVIDIA, Santa Clara, CA, USA) GPU for 100 epochs. The dataset was split into 70% for training, 15% for validation, and 15% for testing. The model was optimized using the Adam optimizer with a learning rate of 0.001.

## 3. Results

**Figure 3:**
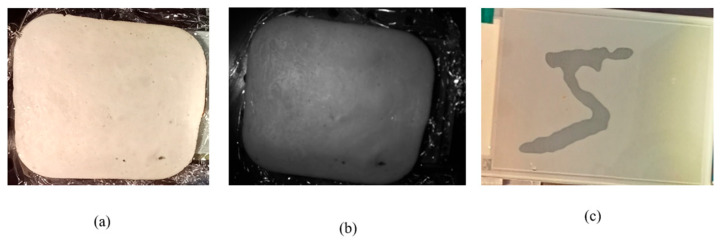
(**a**) Image of a chicken tissue used for our study with a VIS camera. (**b**) Image of the same chicken tissue in the first image captured in the NIR spectrum. Notice that many dark areas appear in the NIR image that do not appear in the VIS image. (**c**) Image of the shape screened underneath the chicken tissue.

The images captured in the NIR band revealed details that are not visible in the visual spectrum (VIS), providing information for the machine learning model that cannot be obtained using a conventional camera, as shown in Figure 3. This observation served as a key validation of the approach, demonstrating the advantage of NIR imaging and motivating its use over VIS for the remainder of the study.

Different tissue thicknesses were evaluated to determine the optimal imaging conditions. A thickness of 5 mm was selected, as it provided a balanced testing scenario: thinner samples revealed too much of the underlying structure, while thicker samples obscured the features almost entirely. Therefore, 5 mm was found to be well-suited for the intended evaluation of the system’s performance.

The illumination was attenuated to reduce glare, in addition to positioning the light source at a 45° angle. Any residual glare effects that persisted under these conditions were intentionally retained, allowing the network to learn and mitigate them as part of the reconstruction process.

The mean squared error (MSE) was employed in our experiments as a metric to quantify the model loss during both the training and validation phases. The loss calculated by the MSE was minimized during the network’s training and validation process. For autoencoder-based neural networks, which form the foundation of the proposed model, MSE serves as a standard loss function for assessing reconstruction performance in regression problems. It computes the average squared deviation between the network’s predicted outputs and the corresponding true values, assigning greater weight to larger discrepancies. The mathematical formulation of the MSE is presented in Equation (2).

(2) $$MSE=\frac{1}{\left(H\cdot W\right)}\sum_{i=1}^{H}\sum_{j=1}^{W}{\left(x_{i,j},-{\hat{x}}_{i,j}\right)}^{2}$$

where *H* represents the image height, *W* is the image width, ***x_i,j_*** is the original pixel value at position (*i,j*), and $\hat{x}$***_i,j_*** is the reconstructed pixel value at the same position in the image.

**Figure 4:**
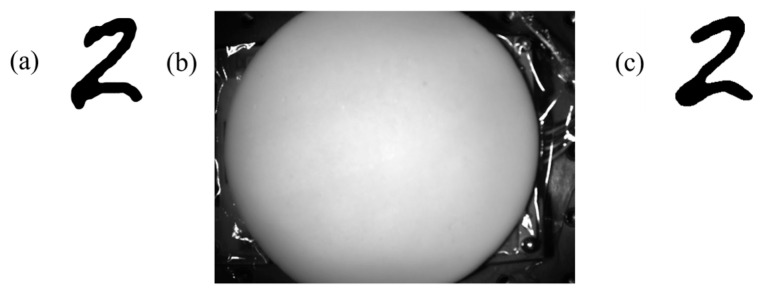
(**a**) The image presented on the screen underneath the object. (**b**) The image of the object taken by the camera (**c**) The image seen in the output of the neural network.

**Figure 5:**
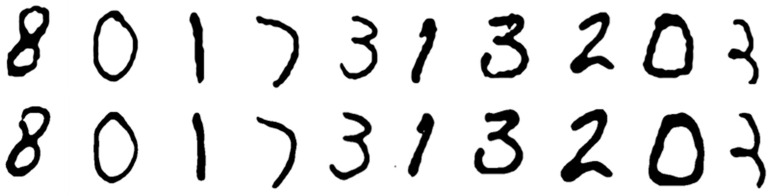
(**Top**): the original images displayed beneath the phantom. (**Bottom**): the reconstructed images, which closely resemble the originals but exhibit smoother edge transitions.

The autoencoder neural network was able to reconstruct the hidden black-and-white digit shapes displayed beneath the object with high visual fidelity. During the testing phase, the model received only the NIR images of the object and successfully reproduced the corresponding hidden shapes that were never shown to it directly, as displayed in Figure 4:

The reconstructed outputs exhibited strong visual agreement with the ground truth images from the MNIST dataset. Some of the reconstructed images appeared almost identical to the original image but showed smoothed edges compared to their original counterparts (Figure 5).

**Figure 6:**
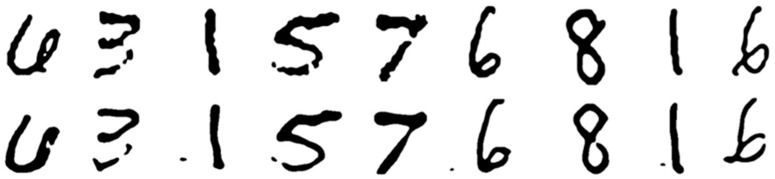
(**Top**): the original images displayed beneath the phantom. (**Bottom**): the reconstructed images, which closely resemble the originals but exhibit slight discrepancies.

Other reconstructed images preserved the overall shape of the original; however, they exhibited minor discrepancies, such as filled gaps, added corners, or minute artifacts that were not present in the original images (Figure 6).

Even though minor differences were observed, the overall structures remained consistent with the original images. Qualitative analysis confirmed that the overall similarity between the original and reconstructed images was preserved.

Image quality assessment metrics such as MSE and Peak Signal to Noise Ratio (PSNR) are commonly used due to their simplicity in calculation, clear physical interpretations, and ease of integration into mathematical optimization tasks. However, these metrics often do not align well with perceived visual quality and lack normalization in their representations. To overcome these limitations, researchers have introduced two normalized reference-based methods that focus on structural and feature similarities between images. The Structural Similarity Index (SSIM) quantifies the normalized structural similarity, while the Feature Similarity Index (FSIM) evaluates the normalized feature similarity between images. Both metrics are full-reference measures, requiring the original image for comparison [26].

For a comprehensive evaluation, 1000 image pairs were created using the original image displayed on the E Ink screen as the reference. The raw NIR images captured through the chicken tissue exhibited a low average PSNR of 4.56 dB, reflecting poor pixel-level similarity to the original E Ink images. Each original image was paired either with its reconstructed output from one of the network models or with the result obtained after applying a Wiener filter to the raw NIR images. PSNR, SSIM, and FSIM metrics were calculated for each pair and averaged across all samples per method. This approach enabled a detailed comparison of reconstruction quality across different network configurations and conventional filtering.

The main U-Net model with a 768-channel bottleneck achieved the highest reconstruction quality, with a PSNR of 20.14 dB, an SSIM of 0.92, and an FSIM of 0.94. The 1024-channel bottleneck variant produced slightly lower results (PSNR = 18.60 dB, SSIM = 0.91, FSIM = 0.91), while the 512-channel two-layer variant achieved intermediate performance (PSNR = 9.57 dB, SSIM = 0.62, FSIM = 0.70). The Wiener filter, applied directly to the raw NIR images, yielded minimal improvements (PSNR = 3.38 dB, SSIM = 0.18, FSIM = 0.51), highlighting the limited capability of conventional filtering compared to neural network reconstructions.

The PSNR was computed with respect to the original black-and-white image displayed on the screen. However, capturing the screen without the tissue reveals that the displayed image is not strictly binary, but contains varying gray levels. Moreover, while the original images are essentially two-level, the reconstructed outputs exhibit a continuous range of grayscale intensities.

Although the PSNR values are relatively low (with optimal values typically above 25 dB), this is primarily due to the pixel-wise nature of the metric and the mismatch between binary ground-truth images and grayscale reconstructions. This discrepancy reduces pixel-wise agreement, even when the underlying structures are accurately recovered. Importantly, the PSNR values remain higher than those of the raw NIR images.

In contrast, SSIM and FSIM remain high, and visual inspection confirms that the main structural features are well preserved. Therefore, the relatively low PSNR does not necessarily indicate poor reconstruction quality, but rather reflects the limitations of the metric for this type of comparison.

These results, summarized in Table 1, demonstrate that the network-based reconstructions effectively recover the original screen content from heavily obscured NIR captures, with the main U-Net model providing the best overall performance.

To directly compare the original E-Ink images with the reconstructed outputs, horizontal cross-sections were extracted and their intensity profiles were plotted. The intensity profiles of both the original and reconstructed images exhibit sharp, binary-like transitions between foreground and background regions, with minimal intermediate gray levels.

This indicates that the network successfully preserved the high contrast and edge sharpness of the original structures without introducing noticeable smoothing artifacts. The reconstructed intensity profiles remain well aligned with the ground truth, accurately reproducing both the position and thickness in each part of the image.

**Figure 7:**
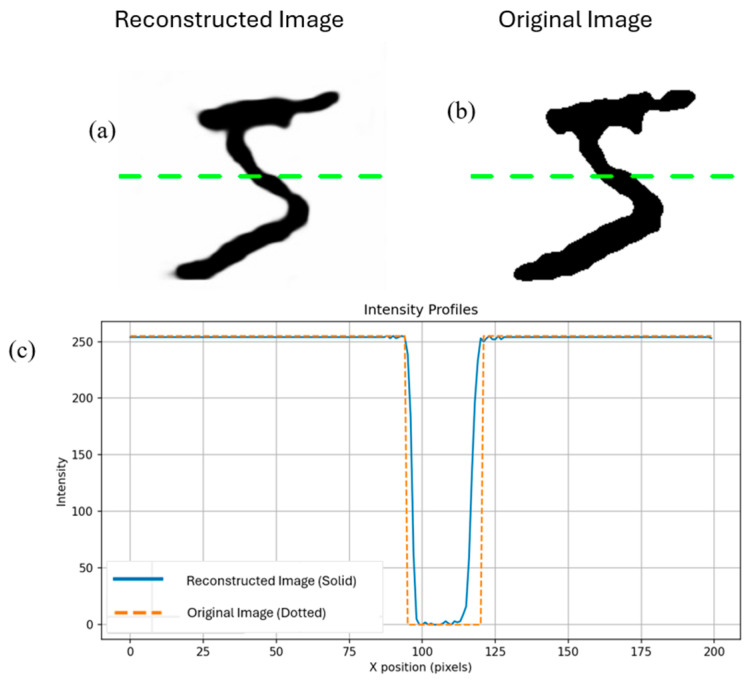
(**a**) Reconstructed image that was processed in the neural network (**b**) Original image (**c**) Intensity graph of the line drawn on both images.

These results confirm that the autoencoder effectively maintains global shape and fine structural details throughout the reconstruction process, as shown in Figure 7.

Out of 1000 images, only five reconstructions failed, with the original digit not discernible. This failure was likely due to factors such as local over-scattering, uneven tissue thickness, or severe blockage of the underlying patterns, which limited the information available to the network. Rare variations in the training data or highly distorted input regions could also contribute to suboptimal reconstructions, highlighting the challenges the model faces with the most difficult NIR captures.

Despite these occasional limitations, the combined qualitative and quantitative analyses demonstrate that the proposed NIR autoencoder system can reliably reveal and reconstruct subsurface structures with high fidelity, even when these features are completely invisible to the naked eye in the VIS.

To assess the generalization capability of the network, NIR images were acquired of modeling clay placed on white paper with a layer of chicken tissue on top, as well as of a square displayed on a screen, independent of the MNIST dataset. The network demonstrated limited performance on these out-of-distribution images indicating partial structural reconstruction and highlighting the need for further optimization.

## 4. Discussion

Our experiment demonstrates that NIR imaging combined with deep learning can recover visual information obscured by scattering tissue. A convolutional autoencoder successfully reconstructed hidden patterns by learning the spectral distortions introduced by the tissue phantom. These results highlight the feasibility of computationally enhanced NIR imaging as a non-invasive imaging strategy.

The experiments demonstrate a certain capability for imaging through scattering tissue; and the observed performance indicates potential, motivating further investigation into whether this approach could be extended to clinical scenarios. Because NIR light is non-ionizing, it enables repeated imaging without the risks associated with ionizing radiation, making it attractive for clinical use. An additional advantage of the proposed system is its operational simplicity. In contrast to many conventional imaging methods that require specialized instrumentation and trained personnel, the system presented here consists of a simple camera connected to a computer and can be operated without complex procedures. The system also provides rapid image acquisition and processing, allowing quick results that may be advantageous during surgical procedures requiring immediate feedback.

However, it is important to emphasize that this technology is not yet clinically ready. The current study was conducted under controlled experimental conditions using simplified targets and tissue phantoms rather than real surgical environments. While the results are promising, they represent a proof of concept rather than a fully validated clinical solution.

In contrast to the simplified experimental configuration used in this study—comprising homogeneous, smooth tissue phantoms with flat embedded targets—a real clinical environment presents substantially greater optical and geometric complexity. Biological tissues exhibit heterogeneous absorption and scattering coefficients that vary both spatially and between patients, and tissue surfaces are typically curved and irregular, leading to angle-dependent reflectance and variable photon path lengths. Furthermore, pathological structures such as tumors may differ in thickness, morphology, and depth, introducing additional variability in attenuation and spectral distortion. Clinical imaging conditions are also dynamic: working distances are not fixed, illumination may involve multiple non-uniform light sources, and physiological motion can introduce temporal fluctuations and motion artifacts. From a computational perspective, these factors represent a significant domain shift relative to the controlled training data used in this study, potentially limiting model generalization. Therefore, future work should incorporate more realistic tissue and tumor phantoms with varying optical properties, geometries, and thicknesses, as well as dynamic imaging conditions, in order to evaluate robustness and advance toward clinically relevant validation.

One practical limitation of the setup concerns the use of a halogen lamp as the NIR source. Halogen lamps generate significant heat, which can alter the optical properties of biological tissue. This issue may be mitigated by operating the lamp in a flash mode—activating it only briefly during image acquisition—thereby minimizing thermal effects. While this approach could be sufficient for short diagnostic procedures, it may be less suitable for prolonged real-time imaging during surgery. Future work should explore alternative NIR light sources, such as NIR diodes, which produce less heat and are more appropriate for continuous clinical use.

Another limitation relates to image resolution. Although the camera resolution was 1440 × 1080 pixels and the E-paper display was 800 × 600 pixels, the U-Net processed resized images of 200 × 200 pixels to enable faster computation. The network reconstructed simple shapes effectively despite this downscaling; however, fine structural details were inevitably lost. Future studies should incorporate larger datasets and higher-resolution inputs to improve robustness, generalization, and the reconstruction of complex, clinically realistic anatomical structures.

Overall, the method offers an efficient and scalable framework for data collection and model training, which is valuable for advancing research in computational biomedical imaging. Nevertheless, substantial validation—including testing under varied illumination conditions, different wavelengths, and on real clinical specimens—is required before this approach can be considered a reliable tool for routine medical practice.

## 5. Conclusions

Overall, this work represents an initial step toward a cost-effective, non-invasive imaging approach, the clinical benefits of which require further validation. The system successfully demonstrated imaging through real biological tissue; however, the current evaluation was limited to images displayed on a screen, which do not accurately represent real-life clinical conditions. In addition, the neural network showed limited generalization capability.

For translation into clinical practice, several improvements are required. First, the dataset must be significantly expanded to enhance robustness and generalizability. Second, systematic testing across different wavelengths and illumination conditions is necessary to determine optimal imaging parameters. Third, validation on actual clinical specimens is essential to assess real-world performance. Finally, reproducibility studies must be conducted under realistic experimental conditions rather than relying on images presented on an electronic display.

Addressing these limitations will be critical for advancing the technology toward practical clinical implementation.

## Figures and Tables

**Table 1:** Quantitative comparison of PSNR, SSIM, and FSIM metrics between chicken tissue and predicted images across 100 samples.

Group	PSNR Mean ± Std	SSIM Mean ± Std	FSIM Mean ± Std
Captured Images	4.56 ± 0.25	0.53 ± 0.04	0.68 ± 0.01
Main U-Net (768-channel bottleneck)	20.14 ± 2.04	0.92 ± 0.05	0.94 ± 0.03
1024-channel bottleneck variant	18.60 ± 2.27	0.91 ± 0.05	0.91 ± 0.03
512-channel two-layer variant	9.57 ± 0.90	0.62 ± 0.03	0.70 ± 0.02
Wiener Filter	3.38 ± 0.19	0.18 ± 0.03	0.51 ± 0.06

## Data Availability

The data are not available at this stage due to time constraints associated with the scope of the current study. Data may be accessed upon request to yossefda@jce.ac.il.

## Abbreviations

The following abbreviations are used in this manuscript:

NIR	Near Infra-Red
AI	Artificial Intelligence
VIS	Visual Spectrum
CNN	Convolutional Neural Network
MNIST	Modified National Institute of Standards and Technology
PSNR	Peak Signal to Noise Ratio
SSIM	Structural Similarity Index
FSIM	Feature Similarity Index
